# Improved neuron protection following cortical injury in the absence of Semaphorin4B

**DOI:** 10.3389/fncel.2022.1076281

**Published:** 2022-12-01

**Authors:** Sahar Sweetat, Natania Casden, Oded Behar

**Affiliations:** Department of Developmental Biology and Cancer Research, The Institute for Medical Research Israel-Canada, Faculty of Medicine, The Hebrew University of Jerusalem, Jerusalem, Israel

**Keywords:** sema4B, astrocytes, neurons, cell death, brain injury

## Abstract

Injury to the central nervous system induces neuronal cell death and astrogliosis, an astrocyte-mediated response that has both a beneficial and detrimental impact on surrounding neuronal cells. The circumstance however, in which astrogliosis improves neuronal survival after an injury is not fully characterized. We have recently shown that Semaphorin4B (Sema4B) in the cortex is mostly expressed by astrocytes, and in its absence, astrocyte activation after an injury is altered. Here we find that in Sema4B knockout mice, neuronal cell death is reduced; as a result, more neurons survive near the injury site. Sema4B protein applied directly to neurons does not affect neuronal survival. In contrast, survival of wild-type neurons is increased when plated on glial culture isolated from the Sema4B knockout mice, as compared to Sema4B heterozygous cultures. Furthermore, this increased survival is also observed with conditioned medium collected from glial cultures of Sema4B knockout mice compared to heterozygous mice. This indicates that the increased survival is glial cell-dependent and mediated by a secreted factor(s). Together, our results imply that following injury, the lack of Sema4B expression in glial cells improves neuronal survival either as a result of reduced toxic factors, or perhaps increased survival factors under these conditions.

## Introduction

Brain damage resulting from stroke or head trauma is one of the leading causes of disability and death in humans. Clinical problems arising following brain injury and traumas are in part the result of neuronal loss. Therefore finding ways to limit this neuronal cell loss is a crucial avenue to improve brain injury outcomes. In addition to the impact on neurons, brain injury is also characterized by the activation of astrocytes adjoining the site of injury. Experimental ablation of astrocytes increases neuronal loss and slows recovery from CNS injury, implying that the presence of reactive astrocytes is crucial under such conditions (Bush et al., 1999; Faulkner et al., 2004). However, manipulation of different signaling pathways in reactive astrocytes demonstrates that astrogliosis might increase or decrease neuronal survival depending on the specific pathways. For example, deletion of STAT3 in astrocytes results in increased neuronal death and more limited recovery following CNS injury (Faulkner et al., 2004; Okada et al., 2006), whereas inhibition of NF-κB in astrocytes improves recovery (Brambilla et al., 2005). It, therefore, appears that activation of astrocytes after injury induces opposing signaling pathways that together determine the degree of neuronal cell death. Thus, identifying additional molecules that regulate different aspects of astrogliosis may have the potential to impact neuronal cell death. One such possible candidate is Semaphorin4B (Sema4B).

Semaphorin4B is a member of the type 4 semaphorins, an evolutionarily conserved family that commonly acts as ligands that bind directly to plexins or neuropilins (Carulli et al., 2021). Recently, we showed that Sema4B is expressed by cortical astrocytes and in its absence, the astrocyte activation profile is altered and proliferation is reduced following cortical injury. We further found that Sema4B is likely to function as a receptor or signaling molecule in astrocytes (Ben-Gigi et al., 2015). These results imply that Sema4B is part of a novel-signaling pathway that regulates astrogliosis after CNS injury. However, whether this altered astrocyte activation can impact neuronal survival is not known. We, therefore, decided to examine the potential of Sema4B to impact neuronal cell death following brain injury.

## Materials and methods

### Animals and surgical procedure

Sema4B heterozygous mice were purchased from the Mutant Mouse Regional Resource Center (MMRRC). This mouse line was generated using targeted trap alleles. In this mouse line, Sema4B is retained within the intracellular compartment (Friedel et al., 2005). A heterozygous breeding strategy was adopted in order to obtain both wild-type, heterozygous (Sema4B±), and mutant (Sema4B−/−) mice. Both sexes were used in the experiments. All animal procedures were performed according to the regulations of the Hebrew university animal care committee. All cortical injury experiments were performed on mice aged 7–8 weeks old. Genotype was determined by PCR analysis of genomic DNA isolated from ear clippings of 3-week-old mice. The presence of the wild-type Sema4B allele was established-using primer 1 (5′-AGACATGGTGCTGGAGAGGT-3′) with primer 2 (5′-TGTGTTTGGTTGGATCTGGA-3′). The mutant allele of Sema4B−/− was verified with primer 3 (5′-TGCACATGCTTTACGTGTG-3′), and primer 4 (5′- TGCCGCGTGTCGTGTTGCAC-3′).

### Stab wound injury model

For the injury experiments, mice were anesthetized with a ketamine/xylazine solution (50 mg/kg ketamine/7.5 mg/kg xylazine in 0.9% NaCl solution). A sterile needle was inserted vertically into the right cerebral hemisphere, reaching the skull surface at a depth of 5 mm. The needle was inserted through the cranium 2 mm caudal to the bregma and 1 mm lateral to the midline. The skin incision was closed with biological glue.

### Immunofluorescence analysis

Brains were fixed in 4% paraformaldehyde, followed by incubation in 30% sucrose for at least 24 h until the tissue sank to the bottom, and then the brains were frozen in OCT (Tissue Tek #4583) in liquid nitrogen. Coronal tissue sections (20 micrometers thick) were fixed again in 4% paraformaldehyde, washed 3 times in PBS for 5 min each, and then incubated for 1 h in a blocking solution consisting of 0.5% Triton X-100, 0.01% NaN_3_, and 5% goat serum. This solution was used for the dilution of both primary and secondary antibodies. Sections were incubated in the primary antibody overnight (16 h). Slides were then washed three times (5 min each) with PBS and visualized with Cy3- and Cy5-labeled secondary antibodies (Jackson Immuno Research Laboratories, Inc., West Grove, PA, United States) and coverslipped with fluorescent mounting media (Dako). Every 5th section was stained and analyzed with the different antibodies. At least seven sections were analyzed for each mouse. The non-injured side was used to determine background levels. Sections were analyzed using a fluorescent microscope (Nikon-TL). Analyses were done using ImageJ software.

### Neuronal survival after stab wound injury

Seven days after injury, 6–8 sections per mouse (100 μm between each section) were stained with anti-NeuN antibody. From the epicenter of the injury, we counted the number of NeuN-positive cells in the area encompassing a distance of 500 microns from each side of the injury and the width of the cortex. The number of cells was normalized to the area measured such that missing tissue parts did not affect the density of cells. The cells were counted using ImageJ (*n* = 8 heterozygous, 7 knockout mice).

### Mixed glia cultures

Mouse primary cortical glia cells were cultured essentially as described previously (Schildge et al., 2013), with minor modifications (Ben-Gigi et al., 2015).

### Conditioned media

For the conditioned medium experiments, when the culture became confluent, the culture was washed 3 times with PBS, replaced with Neurobasal medium, and incubated for 3 days. The conditioned medium was then passed through a 0.22 μm filter.

### Cortical neuronal culture

Plates were coated with poly-d-lysine (Sigma P1024) overnight at 37°C, and cortical neurons were isolated from embryos (E16.5). Cortices were dissected out and the hippocampus, meninges, and pial layers were removed. Digestion solution was added to the cortices [digestion with papain (Worthington biochemical corporation Lakewood, NJ, United States, #3126) 20 units/ml and 2 ml/brain in HBSS] and the samples were incubated for 4 min at 37°C in a water bath. The digestion solution was removed and the same volume of inhibitor solution was added [inhibitor solution: 1 gr trypsin inhibitor (Sigma, T9253) + 1 gr BSA in 1 L HBSS + MgCl + Hepes] and the samples were incubated for 2 min at 37 C in a water bath. The inhibitor was replaced three times. After removing the final round of inhibitor solution, 5 mL of Neurobasal Media (Invitrogen, Waltham, MA, United States, 21103-049) + 1x B27 (Invitrogen, Waltham, MA, United States, 17504-044) were added. The tissues were titrated 10 times with a 1 ml tip. After 1 min, the supernatant was transferred to a new tube and centrifuged for 1 min at 250 g. Then the pellet was suspended with Neurobasal media and the cells were counted and plated on coated plates or on the mixed glial culture. For low density, 100,000 cells per 24 well, and for the Alamar blue assay 40,000 cells per 96 well were cultured. The medium was changed every 3 days. For the experiments with the conditioned medium, the medium was changed to conditioned medium 3 days after culturing.

### Neuronal survival in mixed glial culture using β-3 tubulin staining

A few days after the establishment of confluent glial cultures, neurons were plated and grown for 7 days. After fixation, neurons were stained with anti- β-3 tubulin antibody. To estimate the neuronal numbers, 10 randomly selected fields for each mouse were counted. Each such experiment was carried out 3 times using 3 mice for each genotype.

### Production of secreted protein fusion Fc and western quantification

HEK293T cells were transfected with 20 ug DNA with a 1:2 ratio using Linear- Polyethylenimine reagent (Polysciences, Warrington, PA, USA). The day after transfection the medium was changed to Opti-MEM (ThermoFisher Scientific, Waltham, MA, USA) for 2 days. The concentration of the recombinant proteins was quantitated by Western blot using anti-hIgG secondary Ab. Known quantities of IgG from human serum were used to estimate the Fc fusion proteins. Western protocol (as described later) after blocking for 1 h, one wash with TBST for 5 min, then incubation with Fc Ab (1:1,000 dilution) for 1 h.

Development as described in Western blot protocol. The quantification is against the known IgG concentrations using ImageJ software.

### Fluoro-Jade C staining

Frozen sections were fixed in 4% PFA, washed 3 times for 5 min each with PBS, and dried at 60°C for 30 min. Slides were then immersed in 100% ethanol for 5 min, 70% ethanol for 2 min, and DDW for 2 min. They were then incubated with 0.06% potassium permanganate for 15 min, followed by immersion in DDW for 2 min. They were incubated with 0.0001% FJC (Histo-Chem Inc., Jefferson, AR, USA) in 0.1% acetic acid for 20 min, and again immersed in DDW 3 times for 1 min each. The slides were dried again at 60°C for 5 min and then incubated with Xylene for another 5 min. Finally mounting medium DPX (Sigma #44581) was applied and the slides were covered with coverslips. The staining was analyzed using fluorescent microscopy (Ex 435, Em 525).

### Alamar blue assay

A total of 40,000 neurons per well were plated in a 96-well plate coated with PDL. After 24 h, the medium was removed and the cells were incubated with AlamarBlue^®^ Cell Viability Reagent (Invitrogen, Waltham, MA, United States, DAL1025) for 2.5 h at 37°C. The AlamarBlue was then transferred to a new plate to be read in a fluorometry plate reader (Ex 560, Em 590) On day 7 we repeated the measurement of the same plates and normalized the Optical Density (O.D) at day 7 to day 1. For the conditioned medium experiments, we added the conditioned medium after the first read with the AlamarBlue and changed it every 3 days (the conditioned medium was taken fresh from the culture every time).

### Statistical analysis

Values presented are mean ± SD. A *p*-value < 0.05 was considered significant. Statistical analysis was performed using the one-tailed or two-tailed Mann–Whitney test. Where relevant, *P*-values were adjusted for multiple comparisons in accordance with the Bonferroni procedure; overall *P*-values for the different injury experiments were then computed from these adjusted *P*-values using Fisher’s chi-square test for combined probabilities. Symbols are as follows: *P < 0.05, ^**^P < 0.001, and ^***^P < 0.0001.

## Results

### Neuronal cell death is reduced in Semaphorin4B mutant mice

Brain injury results in varying degrees of neuronal cell death. As described above, astrocyte activation can affect the degree of neuronal survival, and since Sema4B modulates astrocyte activation we decided to examine whether and how injury-induced neuronal cell death is affected in the Sema4B knockout mice.

**FIGURE 1:**
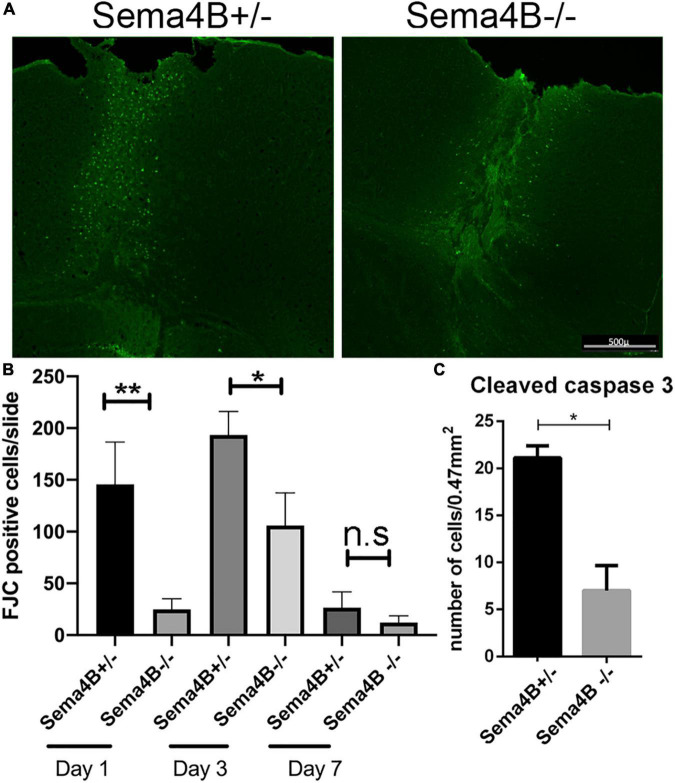
Neuronal cell death is reduced in Semaphorin4B (Sema4B) mutant mice. **(A)** Representative coronal cortical sections in Sema4B heterozygous and Sema4B knockout mice 1 day post-injury labeled with FJC. Scale bar 500 μm. **(B)** Quantification of the total number of dead neurons (FJC positive cells) 1 (*n* = 6, *p* = 0.00408), 3 (*n* = 3, *p* = 0.05), and 7 days post-injury (*n* = 3, *p* = 0.9). **(C)** Quantification of the total number of cleaved caspase three positive cells 1 day after injury (*n* = 3, *p* = 0.05). *P < 0.05 and **P < 0.001.

The peak of neuronal cell death following brain injury takes place 1–3 days after injury (Stoica and Faden, 2010). As a model for penetrating head trauma, we used a stab wound strategy. To detect neuronal cell death we choose to use Fluoro-Jade C (FJC), a fluorescent dye that has been successfully used to label degenerating neurons in the brain (Chidlow et al., 2009). 1 day after the stab wound injury, we found no FJC-positive cells in the contralateral side of the injured brain. In contrast, many FJC-positive cells were detected in the ipsilateral side of the injury. However, in Sema4B knockout mice the number of FJC-positive cells after 1 day was much lower as compared to heterozygous mice (Figures 1A,B). 3 days after injury the difference in FJC-positive cells between Sema4B knockout and heterozygous mice was more modest, although still statistically significant (Figure 1B). After 7 days, only a few FJC-positive cells were detected and no statistical difference between Sema4B knockout and heterozygous mice are detected (Figure 1B).

As an alternative approach to monitoring cell death, we also counted the number of cleaved caspase three positive cells, a well-accepted marker of apoptotic cell death (Eskandari and Eaves, 2022). Since the greatest difference in cell death was 1 day after injury we decided to focus on this time point. Consistent with our FJC results we detected a reduced number of cleaved caspase three positive cells in Sema4B knockout mice as compared to Sema4B heterozygous mice (Figure 1C). Thus, we conclude that neuronal cell death in Sema4B knockout mice is significantly lower than in heterozygous mice.

### Increased neuronal survival in Semaphorin4B mutant mice after cortical injury

**FIGURE 2:**
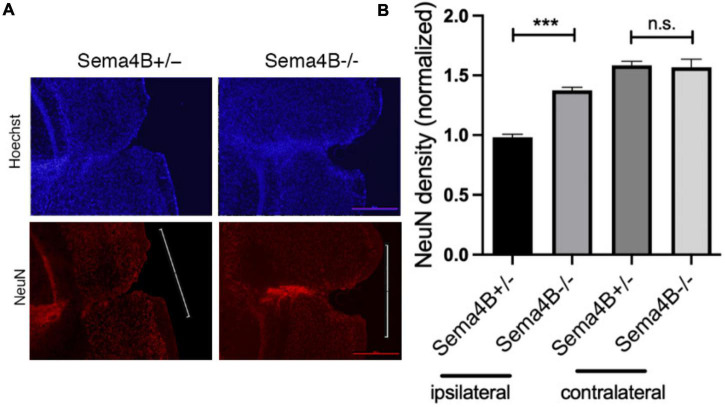
Increased neuronal survival in Semaphorin4B (Sema4B) mutant mice after cortical injury. **(A)** The upper row of the panel shows nuclear stain (Hoechst) in the Sema4B heterozygous and Sema4B knockout mice, and lower row neuronal survival (7 days after injury) was estimated by staining with the anti-nuclear marker specific for neurons, NeuN. **(B)** Results of neuron density in the injured cortex and the non-injured cortex of these same mice (*n* = 7, *p* = 0.00015). Scale bar 250 μm. ***P < 0.0001.

Thus far, we have shown that less neuronal cell death is detected in Sema4B mutant mice after injury. To substantiate this finding and to test whether this reduction in cell death can be attributed to enhanced neuronal survival, we stained for the neuron marker NeuN 7 days after injury. We counted the number of neurons in the cortical gray matter flanking the injury site (500 μM in each direction from the epicenter of the injury). We then calculated neuronal density by dividing this number by the area measured (Figures 2A,B). In Sema4B knockout mice, the density of NeuN-positive cells is about 40% higher than in Sema4B heterozygous mice. The density of neurons in the non-injured contralateral side of both Sema4B ± and Sema4B−/− was as expected higher but without difference between the genotypes (Figure 2B). We therefore concluded that in the Sema4B knockout mice cortex there are more surviving neurons than in the injured cortex of the Sema4B heterozygous mice.

### Increased survival of wild-type neurons co-cultured with glial cells from Semaphorin4B knockout mice

**FIGURE 3:**
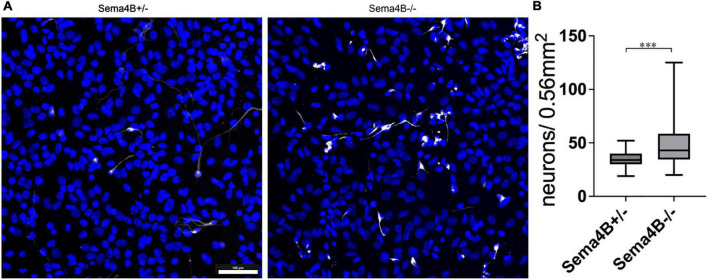
Increased survival of wild-type neurons co-cultured with Semaphorin4B (Sema4B) knockout glial cells. **(A)** Representative image for the co-culture of wild-type neurons in a mixed glial culture from Sema4B heterozygous or knockout mice, 7 days in culture. Cells are stained with the neuronal marker β-3 tubulin (white) and DAPI (blue). (Scale bar 100/m). **(B)** Quantification of β-3 tubulin positive cells after 7 days in co-culture (*n* = 4, ***p < 0.0001).

We have shown recently that Sema4B expression in the brain is mostly restricted to astrocytes. We assumed, therefore, that astrocytes are likely to mediate the increased survival of neurons after injury seen in mice lacking Sema4B expression. Nevertheless, it is possible that other cell types are also involved in neuronal survival. To begin testing this hypothesis directly, we established a mixed glial culture extracted from Sema4B heterozygous and Sema4B knockout mice. We then plated wild-type neurons on top of the mixed glial culture, co-cultured them for 7 days (Supplementary Figure 1) and tested neuronal survival. To estimate neuron survival, neurons were identified by staining with a β-3 tubulin antibody. Interestingly, a significantly higher number of surviving neurons in Sema4b knockout cultures were detected as compared to the Sema4B heterozygous control cultures (Figure 3).

### Neuronal survival is mediated by a secreted factor(s)

**FIGURE 4:**
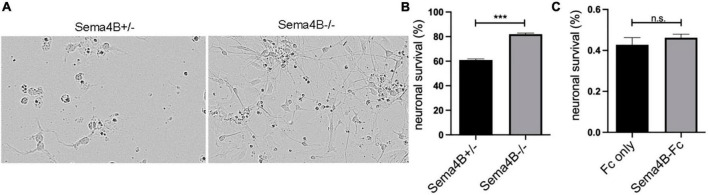
Neuronal survival is mediated by a secreted factor (s) **(A)**. Representative fields of neuronal culture treated with conditioned medium from Semaphorin4B (Sema4B) heterozygous or Sema4B knockout glial cells after 7 days. **(B)** Quantification of the number of surviving neurons that were treated with conditioned medium (*n* = 10, ***p < 0.0001). **(C)** Neuronal survival 7 days after adding the Sema4B-Fc or the Fc-only proteins (*n* = 3, *p* = 0.8).

The increased neuronal survival by mixed glial cultures can be either contact-mediated or alternatively, mediated by secreted factors. To test whether secreted factors are involved we collected conditioned medium from mixed glial cultures prepared from cortices of Sema4B heterozygous and Sema4B knockout mice. Neuronal survival was monitored using the Incucyte^®^ Live-Cell Analysis Systems (Sartorius, Germany) microscopy and followed the same fields before and after the treatment with the conditioned medium. We then counted the cells in order to estimate the degree of neuronal survival in the presence of conditioned medium from mixed glial cultures prepared from Sema4B heterozygous and Sema4B knockout cortices. We observed only 62% ± 0.8 survival of neurons in a conditioned medium from Sema4B heterozygous mixed glia, while in a conditioned medium from Sema4B knockout cultures there was a survival rate of 80% ± 2 (Figures 4A,B).

As an alternative way to test the possibility that secreted factors are involved, we repeated these experiments, but this time neuronal survival was monitored using Alamar blue 1 and 7 days after plating (Supplementary Figure 2). In this method, we also found that the neuronal survival in the culture treated with conditioned medium from Sema4B knockout mice was 2 times higher compared to survival of neurons in culture treated with conditioned medium from Sema4B heterozygous glial culture (Supplementary Figure 2). We, therefore, concluded that the increased survival detected in the absence of Sema4B is mediated to a large degree by a secreted factor(s).

### Semaphorin4B does not interact directly with neurons to influence neuronal survival

Our results so far indicate that in the absence of Sema4B expression in astrocytes, neuronal survival is improved. One possible explanation would be that Sema4B could interact directly with neurons leading to the detected reduced neuronal survival. To test this possibility, we used a secreted version of the Sema4B ectodomain fused to the Fc of human IgG. We then added a concentration of 50 ng/ml of Sema4B-Fc based on our prior study (Birger et al., 2015). As a control, we used 50 ng/ml of Fc-only protein cultured on pure neuronal cultures and monitored neuronal survival after 7 days. We followed the same slide before and after the treatment using the Incucyte^®^ system. In these experiments, 50% ± 7 of the neurons incubated with Fc only survived while 49% ± 5 of the neurons incubated with Sema4B-Fc survived (Figure 4C). We, therefore, concluded that Sema4B does not influence neurons directly.

We validated these findings using an Alamar-blue assay; we cultured cortical neurons and then treated them with Sema4B-Fc or Fc only as a control. We observed 46% ± 0.01 survival when treated with Sema4B-FC and 47% ± 0.02 when treated with Fc only (Supplementary Figure 3). It seems that Sema4B does not interact directly with neurons to influence neuronal survival.

## Discussion

We have previously shown that Sema4B is expressed in astrocytes in the adult mouse cortex and that in the absence of Sema4B expression, astrocyte activation after an injury is altered (Ben-Gigi et al., 2015). However, the significance of this modified activation was not yet clear. Since astrocyte activation is important for neuronal survival, we decided to monitor neuronal survival in the absence of Sema4B following cortical injury. Our results show that in the absence of Sema4B, fewer neurons die and as a result, neuronal survival near the site of injury is improved.

### How does inhibition of Semaphorin4B improve neuronal survival?

In the cortex, Sema4B is mostly expressed by astrocytes and in its absence astrocyte activation is modified (Ben-Gigi et al., 2015). A simple model that could explain the reduced cell death in Sema4B mutant mice would be that Sema4B directly activates cell death pathways in neurons after injury. However, it is not likely that direct action on neurons mediates this effect since the ectodomain of Sema4B added directly to cortical neurons has no effect on their survival. Thus, it is likely that the absence of Sema4B in astrocytes may indirectly affect neuronal survival perhaps by altering astrocyte response to injury. Numerous studies have shown that modulation of astrocyte activation can affect neuronal survival, sometimes increasing neuronal survival. For instance, inhibition of NF-κB, GFAP, or Ndrg2 in astrocytes results in increased functional recovery and increased neuronal survival (Hanbury et al., 2003; Brambilla et al., 2005; Takarada-Iemata et al., 2014). Since our results show increased neuronal survival following injury in Sema4B knockout mice, it is likely that the presence of Sema4B in astrocytes under conditions of brain injury is harmful to neuronal survival.

### How is this effect of Semaphorin4B is mediated?

Our *in vitro* results suggest that the profile of factors secreted by glial cells from Sema4B knockout is better at supporting cortical neurons *in vitro*. This can be the result of increased secretion of survival factors, or alternatively, reduced secretion of harmful factors. Since Sema4B is mostly expressed by cortical astrocytes, it is likely that astrocytes are involved. However, whether or not the improved survival involves other glial or immune cells in addition to astrocytes remains to be determined.

### Can the Semaphorin4B signaling pathway serve as a possible target for injury outcome improvement?

The extent of neuronal cell death following brain injury is a key determinant of the severity of the damage caused to nervous system functionality. Thus, reducing neuronal death is critical in improving the outcome of injury. Using the stab wound injury model, we found that blocking Sema4B results in reduced cell death. However, in the stab wound injury model the degree of cell death is modest, and thus it remains to be seen whether Sema4B is also involved in cell death under more severe traumatic brain injury conditions.

## Data availability statement

The raw data supporting the conclusions of this article will be made available by the authors, without undue reservation.

## Ethics statement

Animal handling adhered strictly to national and institutional guidelines for animal research and was approved by the Ethics Committee of The Hebrew University of Jerusalem, Jerusalem, Israel.

## Author contributions

SS conducted most of the experiments and contributed to experimental design, manuscript editing, and data analysis. NC conducted part of the experiments in Figure 1 and contributed to the manuscript editing and data analysis. OB designed all experiments, wrote the manuscript, and supervised all aspects of the work. All authors contributed to the article and approved the submitted version.
