# Supplementary material for: Improved neuron protection following cortical injury in the absence of Semaphorin4B
Source: Front Cell Neurosci. 2022 Dec 1;16:1076281. doi: 10.3389/fncel.2022.1076281 (PMC9751364; doi:10.3389/fncel.2022.1076281)

Supplementary Figures:

*Figure 1: Representative brightfield image of a coculture of neurons plated on a confluent mix glial culture.*

*
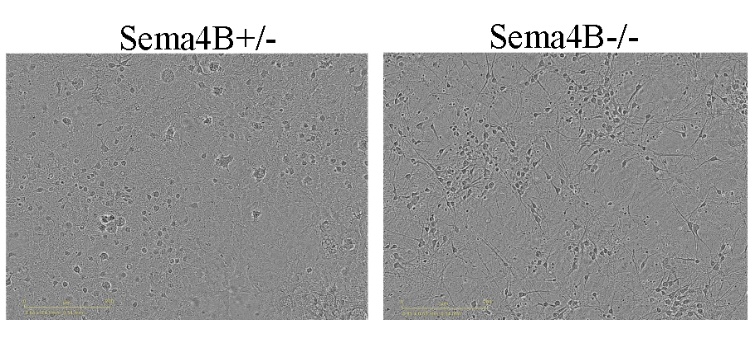
*

*Figure S2: Conditioned medium from Sema4B knockout glial culture better supports neuronal survival*

*Quantifying the number of surviving neurons treated with conditioned medium collected from either Sema4B heterozygous or knockout mixed glial cultures. The neuronal number was estimated on day 1 of plating and 7 days later using Alamar blue (n=3,* P<0.05)


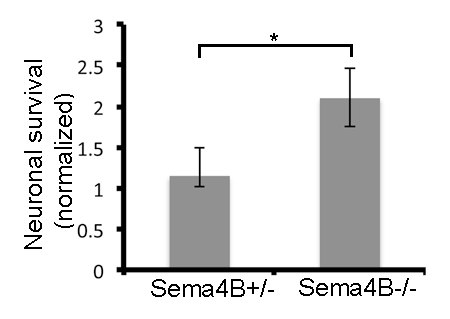


*Figure S3: Sema4B does not induce neuronal cell death*

*Quantification of the number of surviving neurons 7 days after adding Sema4B-Fc or the Fc-only proteins. The neuronal number was estimated at the time of plating and 7 days later using Alamar blue (n=3).*


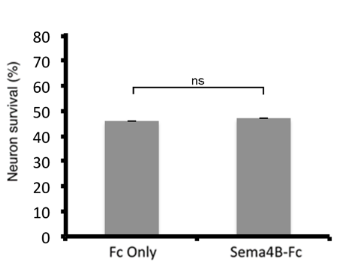

Supplement: Supplementary file 1 [file Data_Sheet_1.docx]
